# Everybody's Page

**Published:** 1890-10-25

**Authors:** 


### Everybody's Page.

The 104th cremation took place at Woking last Saturday.

Owing to the great difficulty of procuring camphor lately, Shanghai housekeepers have been in great trouble as to what measure to take in order to preserve their woollen and fur goods. It is an ill wind that blows no one any good, and the moths must be delighted at the enhanced cost of their deadly enemy. The scarcity has been caused by the hostilities between the aborigines of Formosa and the Chinese settlers.

"Beer should not be drunk out of a glass" is one of the popular science conclusions going round just now. Dr. Schultze says glass is soluble in beer, and lead is used in glass manufacture, therefore beer is injurious. The proportion of lead, the doctor consolingly announces, is very small. So we think, or bottled beer should slay its thousands; so it probably does, but not on account of the lead.

English girls, look to your decorum. Most of the daily papers mention the fact that the Town Council of Treves have opened the municipal gymnasium as a lawn tennis ground for girls in opposition to the wishes of the clerical minority. It is a game unbecoming to German girls say these worthies. We are glad fate has not ordered our destiny to live in Treves with such practical sanitarians as these clericals to order our goings. The *Globe* says the only way in which it can account for the clerical disapproval is that they must have misapprehended the meaning of "underhand service"!

The New York *Medical Record* quotes some astonishing remarks from a Cincinnati medical contemporary as to the way practitioners should proceed to "get on." One piece of advice is "A patient should always be kept waiting a few minutes; it calms agitation, and leads them to think you have another client in your private office." "Console and reassure a patient, leading him to always hope for a cure, even though you know the malady to be incurable." "Most people who visit a doctor desire to be told that they are ill; to tell them the contrary is to make them out asses." "The men with the largest practice are the patient listeners and greatest liars." If these are meant for witticisms, that Cincinnati journal might have said so in a footnote.

At Cardiff last week a milk vendor was sentenced to pay £5 and costs, or go to prison for a month with hard labour, for the offence of selling milk at his house while the inmates were suffering from diphtheria. It seems to us that for this offence, and similar ones, a fine is an inadequate punishment. A man deliberately risked the lives of hundreds of his fellow-creatures in order to save himself a little extra trouble, and he gets off with five pounds fine. He counts it as five pounds loss, and the effect it has on him and others is of a very transitory nature. The sympathetic point out that a month's imprisonment means ruin, but then the milkman causes death by his illegal proceedings. A few more punishments would show that milk-vending has enormous responsibilities, which are not to be lightly overlooked to the danger of an entire neighbourhood.

The numbers and consequent competition of doctors are said to be as great in the Australian colonies as they are in Europe or America.

An American paper deprecates the waning use of a habit called the "spank cure." Children now, when they are just entering that period of their lives when "spanks" are salutary and necessary, are the possessors of "nerves," and so have to be humoured. "The family physician," says the stern writer, "is called in when an application of the slipper is all that is necessary."

Mr. Hugh Alexander presided on Saturday at the Association of Sanitary Inspectors, and attention was called to the fact that the necessity of statutory definition of the qualifications for sanitary inspectors, their duties and salaries, etc., was very great, and wanted settlement, as at present the manner in which sanitary inspectors discharged their duties was inimical to public health.

Children suffer very considerably from thirst, very often, thanks to an ignorant idea of their nurses that water is bad for them. Perfectly pure cold water, given in reasonable quantities, would often stop the over-physicking of children which is carried on at present. The fact is that too many mothers take no intelligent care in dieting their children; they seek no why or wherefore, and prefer to dose rather than avoid the necessity of dosing.

The tendency of public opinion in the direction of paying more attention to hygiene is shown by the passing of various Acts bearing on public health during the past few years. But we quite agree with Dr. Newsholme that Acts of Parliament have an unfortunate knack of remaining dead letters unless they are cordially supported by the local authorities, who should put them in motion. His suggestion that hygiene should be taught in elementary schools—not such as to make every man his own doctor—has much in its favour. Yet, before this can be done, it will be necessary to find teachers competent to impart the necessary instruction. The supply of such teachers is, at present, decidedly limited.

A report has recently been drawn up by the Government statistician upon the meat supply of Australia, which is not without its disquieting features so far as the supply of beef is concerned. The increase of cattle has not kept pace, it appears, with the rate of increase in population in New South Wales and Queensland, and the compiler of the report calculates that, under existing conditions, the present rate of consumption will, in the course of the next eleven years, overtake the supply. With sheep the prospect is brighter, and it is estimated that seventy-four years will probably elapse before consumption overtakes the supply. That there should be any danger of a serious diminution in the food supply of the country is not surprising, when we are told that among the colonists the consumption of meat per head amounts to 276 lbs., as against 105 lbs. per head in this country.

